# A scanning probe microscope for magnetoresistive cantilevers utilizing a nested scanner design for large-area scans

**DOI:** 10.3762/bjnano.6.46

**Published:** 2015-02-13

**Authors:** Tobias Meier, Alexander Förste, Ali Tavassolizadeh, Karsten Rott, Dirk Meyners, Roland Gröger, Günter Reiss, Eckhard Quandt, Thomas Schimmel, Hendrik Hölscher

**Affiliations:** 1 Institute of Microstructure Technology, Karlsruhe Institute of Technology (KIT), Hermann-von-Helmholtz-Platz 1, 76344 Eggenstein-Leopoldshafen, Germany; 2 Institute of Nanotechnology, Karlsruhe Institute of Technology (KIT), Hermann-von-Helmholtz-Platz 1, 76344 Eggenstein-Leopoldshafen, Germany; 3 Institute for Materials Science, Christian-Albrechts-Universität zu Kiel, Kaiserstraße 2, 24143 Kiel, Germany; 4 Department of Physics, Bielefeld University, Universitässtraße 25, 33615 Bielefeld, Germany; 5 Institute of Applied Physics, Karlsruhe Institute of Technology (KIT), Wolfgang-Gaede-Straße 1, 76131 Karlsruhe, Germany

**Keywords:** atomic force microscopy (AFM), magnetomechanical effects, magnetostriction, scanning probe microscopes and components

## Abstract

We describe an atomic force microscope (AFM) for the characterization of self-sensing tunneling magnetoresistive (TMR) cantilevers. Furthermore, we achieve a large scan-range with a nested scanner design of two independent piezo scanners: a small high resolution scanner with a scan range of 5 × 5 × 5 μm^3^ is mounted on a large-area scanner with a scan range of 800 × 800 × 35 μm^3^. In order to characterize TMR sensors on AFM cantilevers as deflection sensors, the AFM is equipped with a laser beam deflection setup to measure the deflection of the cantilevers independently. The instrument is based on a commercial AFM controller and capable to perform large-area scanning directly without stitching of images. Images obtained on different samples such as calibration standard, optical grating, EPROM chip, self-assembled monolayers and atomic step-edges of gold demonstrate the high stability of the nested scanner design and the performance of self-sensing TMR cantilevers.

## Introduction

Since its invention in the 1980s [1] the atomic force microscope (AFM) became a versatile tool frequently used in nanoscale metrology, biosensing, maskless lithography and high density data storage with nearly as many sensing techniques as applications [2–5]. Current state of the art instruments use micro-fabricated silicon and silicon-nitride cantilevers with an optical read-out [6] and image with high resolution down to the atomic scale. Furthermore, AFMs are often incorporated into quality control systems for the fabrication of micro- and nanostructures, especially for industrial applications. For these applications, not only a high resolution, but also a large scan range (field of view) and a compact instrument design of the read-out is desirable [7].

However, most AFMs feature only a limited scan range of typically tens of micrometers. Unfortunately, it is not possible to expand the scan range by simply scaling the instrument dimensions because of the limitations of piezo actuated scan stages commonly used in AFMs. While piezo scanner stages have huge advantages in terms of dynamic properties and smoothness of motion in comparison with motorized stages, their maximum extension remains limited to hundreds of micrometers by using mechanical levers for motion amplification. Additionally, a large scan range and a high lateral resolution are contradictory. Because of these challenges, previous attempts to realize a high resolution and a large field of view use multiple scanning tips recording individual images and a stitching thereof [8] or a combination of motorized large scan range stages with a fast piezo to compensate for the poor dynamics of such stages [9]. In this work, we applied a different approach and nested a small high resolution scanner on a large piezo scan stage enabling both, a large scan range of 800 × 800 × 35 μm^3^ and a high resolution capable of imaging subnanometer features.

The instrument is equipped, like most state-of-the-art instruments for ambient conditions, with an optical read-out of a micro-fabricated cantilever [10–11]. However, the optical read-out contains bulky mechanical parts to focus a laser on the backside of the cantilever and to move the position sensitive photodetector (PSD) or a mirror which puts severe limits on a compact instrument design. Additionally, while adjusting the laser and photodetector is straightforward under ambient conditions under which all components are accessible, it is a challenge in environments such as vacuum or in fluids where the laser light gets scattered and refracted by multiple interfaces [12–15]. Furthermore, optical read-outs have to be readjusted not only after every cantilever exchange but also after temperature drifts which can offset the focal position of the laser and photo-detector due to thermal expansion. Additionally, the optical read-out can influence the cantilevers deflection by photothermal excitation [16] and interfere with the sample as it can cause photobleaching of fluorescence samples [17]. For specific applications and environments like vacuum, self-sensing tuning forks with manually attached tips can greatly simplify instrumentation but at the cost of reduced operation modes [18–20]. Micro-machined cantilevers on the other hand are more versatile and can be mass-produced [21]. Additionally, cantilevers produced by silicon-based microfabrication methods allow for the integration of multiple additional features such as doping for better electrical conductance or the integration of active sensing elements. Previous works incorporated piezo-electric layers [22–23], piezo-resistive layers [24–29] into such cantilevers or added a capacitive readout [30–31] to measure the cantilevers deflection, however, they suffer from a reduced sensitivity compared to the optical read-out. Magnetic sensors [32–34], especially strain sensors based on tunneling magnetoresistive (TMR) junctions [35] had recently shown an enhanced sensitivity compared to piezoresistive sensors [36–40] and are promising candidates for strain sensors incorporated into AFM cantilevers. The instrument presented here has been optimized for the characterization of such self-sensing TMR cantilevers. The microscope is fabricated entirely from non-magnetic materials in order to minimize the instruments influence on magnetic fields which at present are needed to bias the TMR sensors and set their sensitivity at maximum for imaging atomic step edges.

## Setup of a nonmagnetic large scan range AFM

In order to characterize magnetoresistive strain sensors integrated into AFM cantilevers, the deflection of the cantilever has to be measured in parallel by independent means. Therefore, our AFM is equipped with an optical beam deflection setup to measure the deflection of the cantilever [10–11]. This setup also allows for the use of conventional silicon and silicon nitrite cantilevers using only the optical beam deflection setup for the feedback. Additionally, the instrument is designed to apply an external in-plane magnetic bias fields, as the strain sensitivity of TMR sensors used in this work strongly depends on their magnetic configuration. This constrained requires a setup in which coils can be integrated for the application of a bias field. The magnetic field has to be homogeneous and should not interact with the materials used to build the instrument.

**Figure 1:**
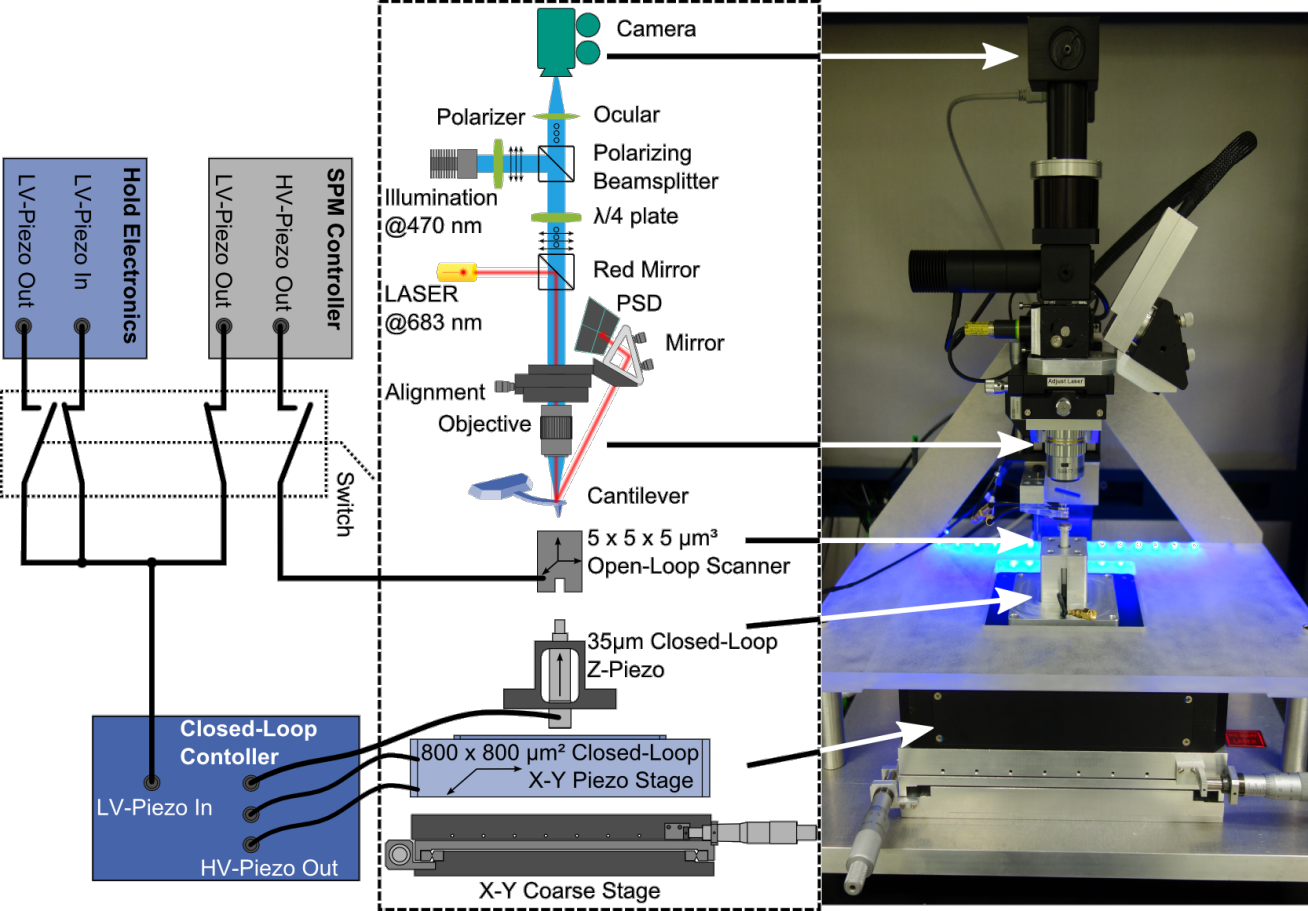
Schematic and photo of the setup including the optical beam deflection and the nested scanner design. The long-working-distance objective of the reflected light microscope is used to focus the laser on the rear side of the cantilever while the reflected beam is focused and reflected on a PSD with a tilting mirror for alignment. To realize both high lateral resolution and large-area scanning, a high resolution open loop scanner is nested on a large-area scanner. For switching between both scanners, the large-area scanner can be held on any position by feeding a constant control voltage to the closed loop controller while small-area scans are performed by the open loop scanner.

The optical beam deflection setup has been integrated into an optical microscope that is used to focus the laser spot on the cantilever. By using a long working distance objective, the beam deflection setup is placed outside the coils for the external magnetic bias field. The optical setup is shown in detail in Figure 1. The use of an infinity-corrected microscope objective and an ocular lens allows one to illuminate the sample and to focus the laser beam on the cantilever with the same objective. Using the microscope objective to focus the laser also simplifies the adjustment of the laser beam deflection setup because the complete optical microscope can be moved instead of adjusting the laser. As a result, the focal spot of the laser is fixed towards the field of view of the optical microscope and the laser is aligned to the cantilever when the cantilever is at a specific position in the optical image. To block scattered light inside the optical path of the laser from the camera, a red mirror is used to couple the laser beam into the objective. As the mirror reflects only light with wavelengths longer than 600 nm, all light from the laser is either reflected towards the objective or the laser itself. The cantilever is tilted towards the optical axis of the microscope and acts as a mirror for the laser beam. As the cantilever gets deflected, the angle of the cantilever tilts towards the incident laser beam and consequently the reflection angle changes. As the reflected beam is divergent (due to the focusing of the microscope objective), it is refocused by using a lens and reflected to the position sensitive photo-detector by a tilting mirror.

To illuminate the sample, a wavelength shorter than the reflection edge of the red mirror was chosen. To suppress stray light within the optical path of the microscope, it is useful to use polarizing optics. In contrast to the laser, the light from the illumination source reaches the sample, is then reflected off the sample, and the reflected light must pass through the complete microscope to reach the camera. By using polarized light for illumination, a polarizing beamsplitter can be used to reflect all light from the light source of the illumination towards the sample. By passing a λ/4 plate, the polarization direction gets rotated by 45°. After being reflected on the sample, the light passes the λ/4 plate again and the polarization is rotated again by 45°. The polarization of the reflected light is now rotated by 90° towards the incident light from the light source. Therefore, the beamsplitter is completely transparent for light reflected from the sample, which can pass towards the camera.

The AFM is operated through a commercial AFM controller (Asylum Research). The controller can directly drive open-loop piezo scanners, because of its integrated high-voltage amplifier, as well as closed-loop scanners with an attached high voltage amplifier and closed-loop controller, as it is also equipped with a low-voltage piezo-drive output. To drive two scanners independently, external control electronics are attached to the controller, which allow for a switching between the high- and low-voltage output. Additionally, this electronics allows one to hold the low-voltage output at any level when switching from the low-voltage output to the high-voltage output and vice versa. As our AFM setup is equipped with two independent scanners to combine both, a large field of view and a high spatial resolution, these hold electronics allow to drive the small-area scanner directly while holding the large-area scanner at a fixed position. The high resolution open-loop scanner is thereby mounted on a large-area scan stage. The high resolution scanner was realized by using a stack of shear actors for x–y scanning and a stack piezo actor with a travel of 5 μm and a resonance frequency of 50 kHz each. The large-area scanner on the other hand is a combination of an x–y piezo large-travel scan stage and a preloaded piezo stack actor for the z-axis. The large travel is achieved by piezos with a comparable small travel pushing a lever to enhance the stage travel, a principle that is typically called lever motion amplifier. For a large-area scanner, lever motion amplification is a suitable way to reach large travels due to certain constraints, although a lever-motion-amplified piezo stage commonly shows a higher noise level than a directly driven stage. The elongation of a piezo is approximately Δ*L* = ±*E*·*d*·*L*_0_, where *E* is the applied electric field, *d* the piezoelectric coefficient of the material and *L*_0_ the initial length of the piezo with typical values for piezo stack actuators of *U* = ±220 V, *d* = 350 pm/V and a distance between two electrodes of 1 mm. To achieve a travel of 800 μm by direct drive, approximately 1 m of piezo ceramic per axis would be required. Such large piezo stacks, however, are neither commercially available nor mechanically stable enough for such a large-area scan stage. However, this design has a reduced mechanical stiffness and resonance frequency.

The reduced resonance frequency increases the response time of the scanner to driving signals. Therefore, lever amplification can only be used for the slow lateral scanning as the z-axis of the scanner needs a high resonance frequency for a fast response to driving signals. The large-area scanner has a motion-amplified x–y piezo stage and a dedicated z-piezo for a short response time. Additionally, the x–y stage must only move in the x–y plane without any cross-talk to the z-axis. This is reached by flexure joints. However, as the stiffness of a lever amplified system is reduced quite significantly, the initial stiffness of the flexure stage has to be quite high. A custom-built scanning stage fulfilling those requirements was therefore developed specifically for this application. Because of the stiff flexure joints, each axis of the stage is equipped with two piezos in parallel movement to increase their pushing force. The piezo elongations and the stage position are each monitored with a capacitive positioning sensor which allows for a linearization of the stage movement by an additional stage controller. The z-piezo of the large-area scanner is a piezo stack with a maximum travel of 35μm and a resonance frequency of 14 kHz while carrying the open-loop scanner. For closed-loop operation of the AFM, this piezo is equipped with a strain gauge sensor which is read out by the AFM controller.

## Results and Discussion

### Characterization of the microscope

**Figure 2:**
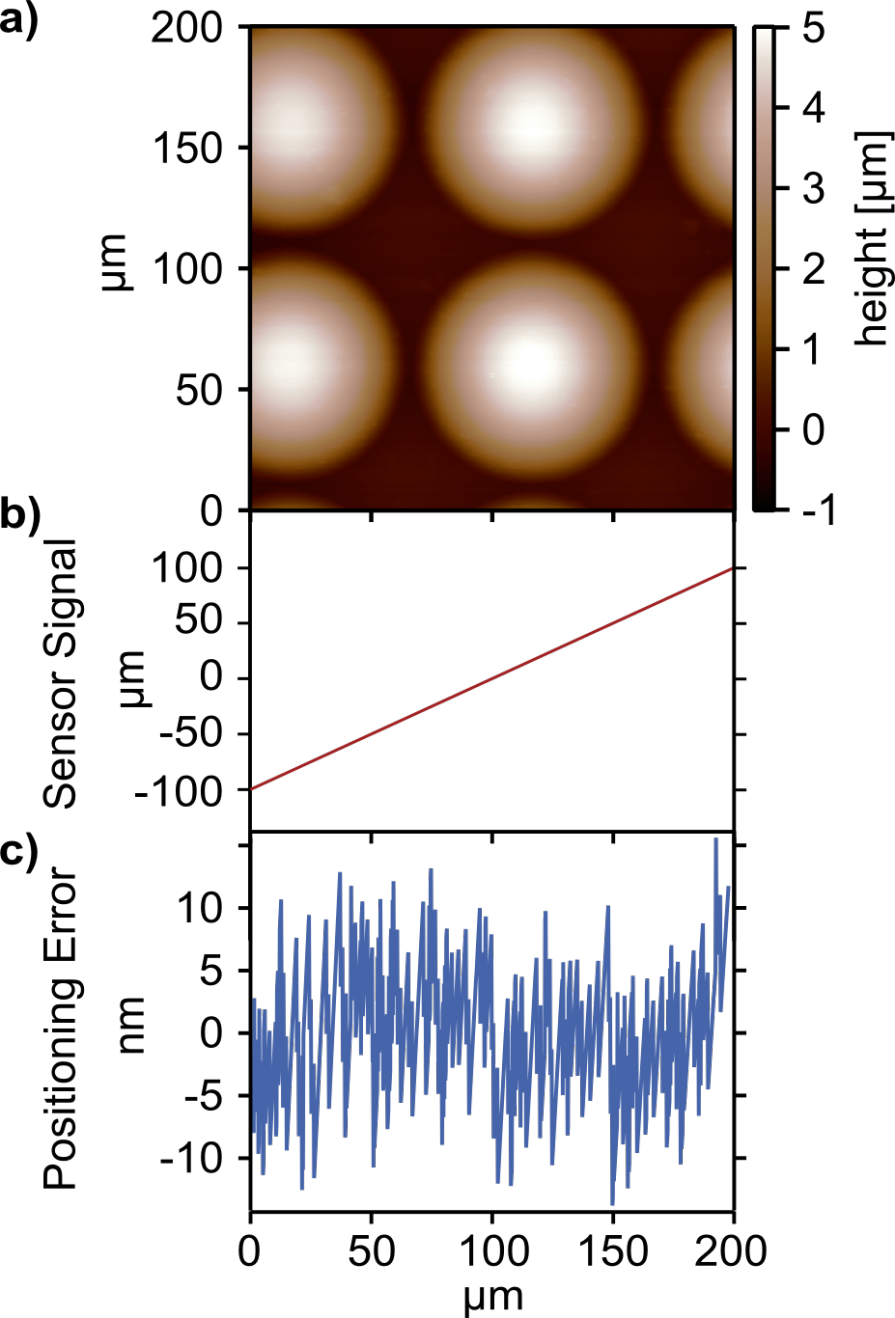
A crucial precondition for a nested high resolution scanner design is the stability of the housing large-area scanner. The position accuracy and positioning error can be tested by reading out the sensing elements for the closed-loop system. a) While scanning the topography of microlenses, b) the read-out of the closed loop sensor in the fast scan direction is recorded. c) The read-out of the fast scan axis is compared with the desired scan position and a positioning error is extracted. The positioning error is below 10 nm for typical scan frequencies. The sensor is read-out with a sampling rate of 1.5 kHz.

For successful switching from the large scanner to the nested scanner, the stability of the large-area scanner has to be high. The positioning accuracy can be tested during AFM scanning. If scanned with the open-loop scanner, also the stability and drift of the large-area scanner is of interest. In Figure 2a, a scan of polymeric microlenses is shown when using the optical beam deflection setup for the feedback. In parallel, the positioning error (profile after removing the 1st order component) of the fast scan axis was recorded and is shown in Figure 2c. By comparing the measured stage position and the desired position (given by the control signal), the positioning error was extracted. The data shows no drift of the stage during the whole experiment and only small fluctuations around the desired position of ±10 nm, which is a low value for a scan stage that has a maximum travel distance of 800 μm.

**Figure 3:**
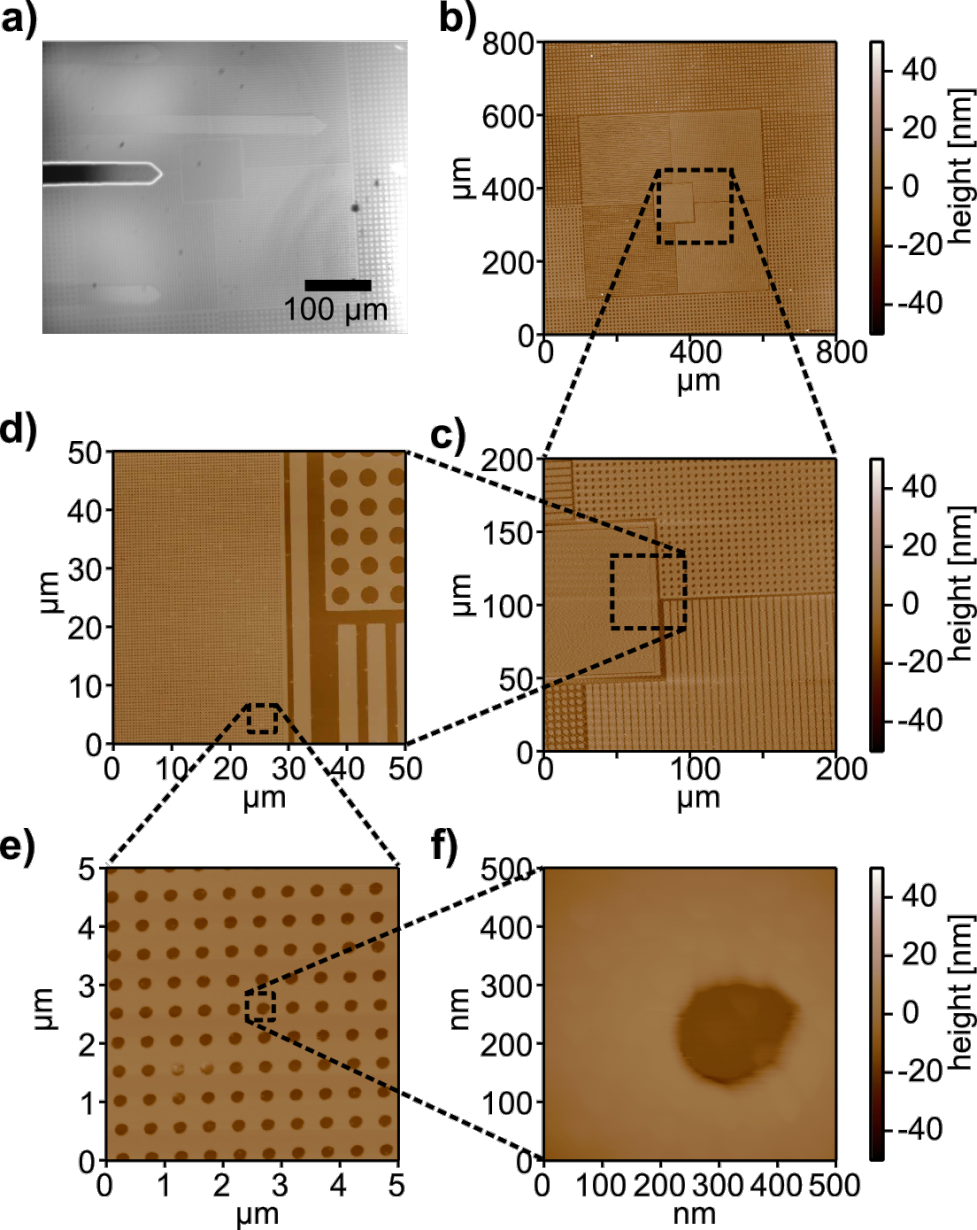
a) Optical microscopy image of a SiO_x_ calibration grating with various feature sizes. Demonstration of large-area AFM imaging with switching to the small-area scanner for high resolution. b) First, an 800 × 800 μm^2^ overview image of the structure was taken. c) Afterwards, the scansize was reduced to 200 × 200 μm^2^. d) The scansize was further reduced to 50 × 50 μm^2^ before switching to the high resolution scanner. e) After switching, 5 × 5 μm^2^ and f) 500 × 500 nm^2^ images of the smallest feature sizes of the calibration grating were taken. The nested scanner design which can span over three orders of magnitude in scan range makes this instrument a versatile tool for micro- and nanomechanical analysis.

As the large-area scanner is mechanically stable, it can be used to carry a second small-area scanner with a higher spatial resolution and better dynamic properties. Using an AFM with multiple scanners allows for both, a large field of view and a high spatial resolution. By using the optical beam deflection setup as well, the potential of such an instrument is demonstrated in Figure 3. By scanning a calibration structure with feature details spanning from hundreds of micrometers to less then 200 nm and a feature height of 22 nm, the topography of the sample can be investigated on all length scales. For a first overview of the sample, the maximum scan size can be used (Figure 3b). Afterwards, sequential zooms into the region of interest are possible (Figure 3c–e). As the desired zoom level results in a scan size below the maximum scan range of the high resolution scanner, the scan position can be held with the large-area scanner while the sample is scanned with the small-area scanner enabling further zoom steps (Figure 3f). Thereby, the instrument can span over three orders of magnitude in scan range, which makes it a helpful tool for micro- and nanomechanical analysis.

**Figure 4:**
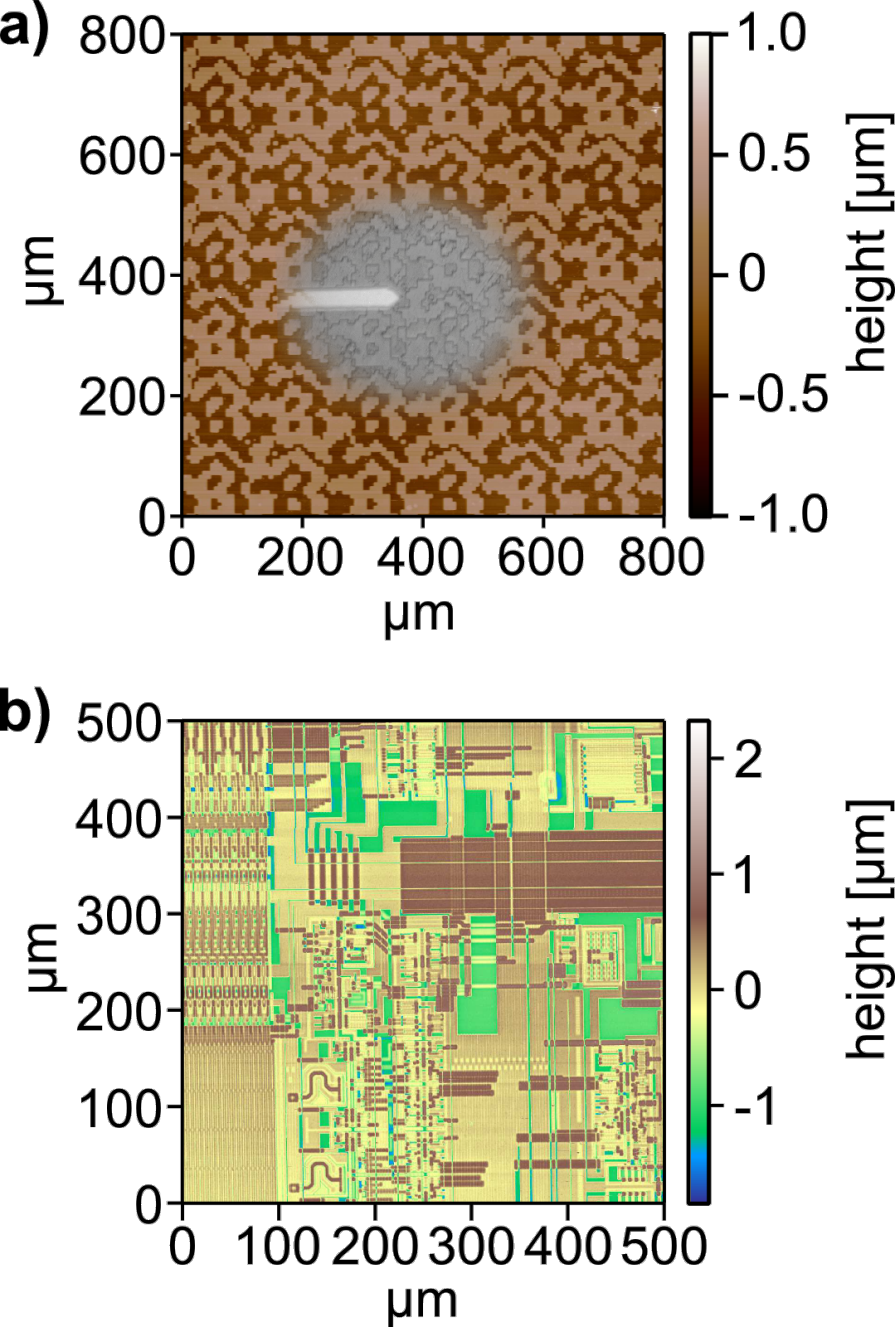
a) Overlay of the optical microscope image with the AFM topography of an optical grating structure with a 256 × 256 μm^2^ grating periodicity. b) Large-area AFM topography image of a part of a UV-erasable CMOS EPROM memory chip with a scan size of 500 × 500 μm^2^. Despite the large step heights of 2 μm, there no artefacts are visible, no loss of contact happened and the features of the chip architecture are clearly visible. The heights of the different layers are constant over the whole scan area which allows for reliable absolute topography measurements in combination with a fast overview using the optical microscope.

One example of such an analysis is given in Figure 4a. For quality control of fabrication steps in microstructure technology, AFMs are often used for spot checks of the fabricated structures. However, as most AFMs are limited to a field of view of 100 × 100 μm^2^, they are only suitable for local imaging. Often, features of structural details will just not fit into this field of view. Optical phase gratings are an example for this type of samples [41]. Imaging such structures with the large-area scanner allows one to image multiple grating periods of 256 × 256 μm^2^ in a single AFM picture and to overlay them with the optical microscope image obtained during scanning. Such diffractive structures define the length of the optical path of the light propagating through by their topography. At least one grating period has to be imaged in order to characterize such grating structures which requires a large scan range. An other challenge are high steps in micro- and nanostructures. However, in many cases, the simultaneous investigation of small features such as transistors (on the nanometer or sub-micrometer scale) and much larger features such as chip architectures (on the millimeter scale) have to be imaged. An example of such structures are microelectronic integrated circuits. Imaging such structures with a special large-area scanning AFM allows for inspection of a wide field of the chip architecture within one scan. Figure 4b shows a portion of the die surface of a UV-erasable CMOS EPROM memory chip (Type 27C256). The image size is 500 × 500 μm^2^ imaged with a resolution of 1024 × 1024 pixels. Imaging was done in the intermittent contact mode of the AFM with a setpoint of 89% of the free amplitude of the cantilever. Due to the large step heights of up to 2 μm on the surface of the chip, and the corresponding high demands on the z-feedback loop the scan speed was set to 30 μm/s. The image shows the original raw data, exhibiting no artefacts or defects and despite the relatively soft tapping and large step heights, no loss of contact to the surface occurred over the whole scan area. All elements on the chip are clearly discernible. Due to the hardware-linearized scan and the very low thermal drift of the setup, no further image processing was necessary. The choice of color table allows for a clear distinction of the different layers of which the circuit is comprised. This shows that also the height scale measured by the AFM is constant over the whole large scan area, a key requirement for reliable quantitative large scale AFM measurements.

### Magnetoresistive strain sensors

Driven by the increasing demand for magnetic hard disk drives [42], magnetic tunneling junctions (MTJ) [43–50] are state-of-the-art read-heads in magnetic hard drives. Additionally, they can be adapted for high strain sensitivity [51] and offer remarkable miniaturization opportunities [52]. In combination with already implemented processes of mass production, they are a promising alternative to piezoresistive and piezoelectric sensors for self-sensing AFM cantilevers [23]. Therefore, we used such magnetic tunneling junctions with magnetostrictive electrodes deposited and patterned on Si substrates as strain sensor on AFM cantilevers. The Si substrates were structured into AFM cantilevers by means of microelectromechanical systems (MEMS) technology [35]. The magnetic tunneling junction consists of two ferromagnetic CoFeB-electrodes separated by a thin dielectric MgO layer, which acts like a spin-valve. The electrical conductance of the magnetic tunnel junction, therefore, strongly depends on the orientation of the magnetization of the electrodes towards each other. When magnetostrictive materials are used in the electrodes [53], the magnetization of one electrode can rotate if strained because of the inverse magnetostrictive effect [54]. To use this effect for strain sensing, only the magnetization of one electrode must rotate when strain is applied to the junction while the magnetization of the other electrode should remain in its initial orientation. Therefore, the MTJ has to be integrated into a TMR stack, which includes contact electrodes and a pinning mechanism to fix the magnetization of one reference electrode while the second sense electrode is free to rotate. To fix the magnetization of the reference layer, it is magnetically pinned by a 0.9 nm thick Ru layer to a CoFe layer by an antiferromagnetic interlayer coupling. The exchange bias between a natural antiferromagnet (IrMn) and the CoFe then fixes the magnetization of the reference layer. Then, the resistance of the tunneling junction varies by rotating the magnetization of the free sensing layer. Using the inverse magnetostrictive effect in the sensing layer makes the TMR stack sensitive to applied strain.

We used a CoFeB (3 nm)/MgO (1.8 nm)/CoFeB (3 nm) TMR junction with an MnIr (12 nm)/CoFe (3 nm) exchange bias system that was annealed at about 360 °C for 1 h at a pressure of 10^−6^ mbar under a magnetic field of 2 kOe for a crystallization of the CoFeB electrodes and improvement of CoFeB/MgO interfaces. It also aligns the easy axis of the sensing layer and pins the reference layer due to the imposed magnetic exchange bias [55]. The TMR stack is grown by sputtering techniques on a 4'' Si(100) wafer substrate with 300 ± 2 μm thickness (Si-Mat Silicon Materials, Germany) with thermally grown 2 μm-thick and 100 nm-thick SiO_2_ layers on the rear and front side, respectively. The TMR sensor AFM cantilevers are prepared by a sequence of MEMS techniques including photolithography, reactive ion etching (RIE), ion beam etching (IBE) and wet etching. The cantilevers used in this study were 300 to 350 μm long and 40 μm wide. To ease the fabrication process thicknesses ranging from 10 μm to 20 μm were chosen. The resulting resonance frequencies of the cantilevers vary from 170 kHz to 270 kHz and their spring constants from 40 N/m to 440 N/m.

### Measurements with TMR sensors

**Figure 5:**
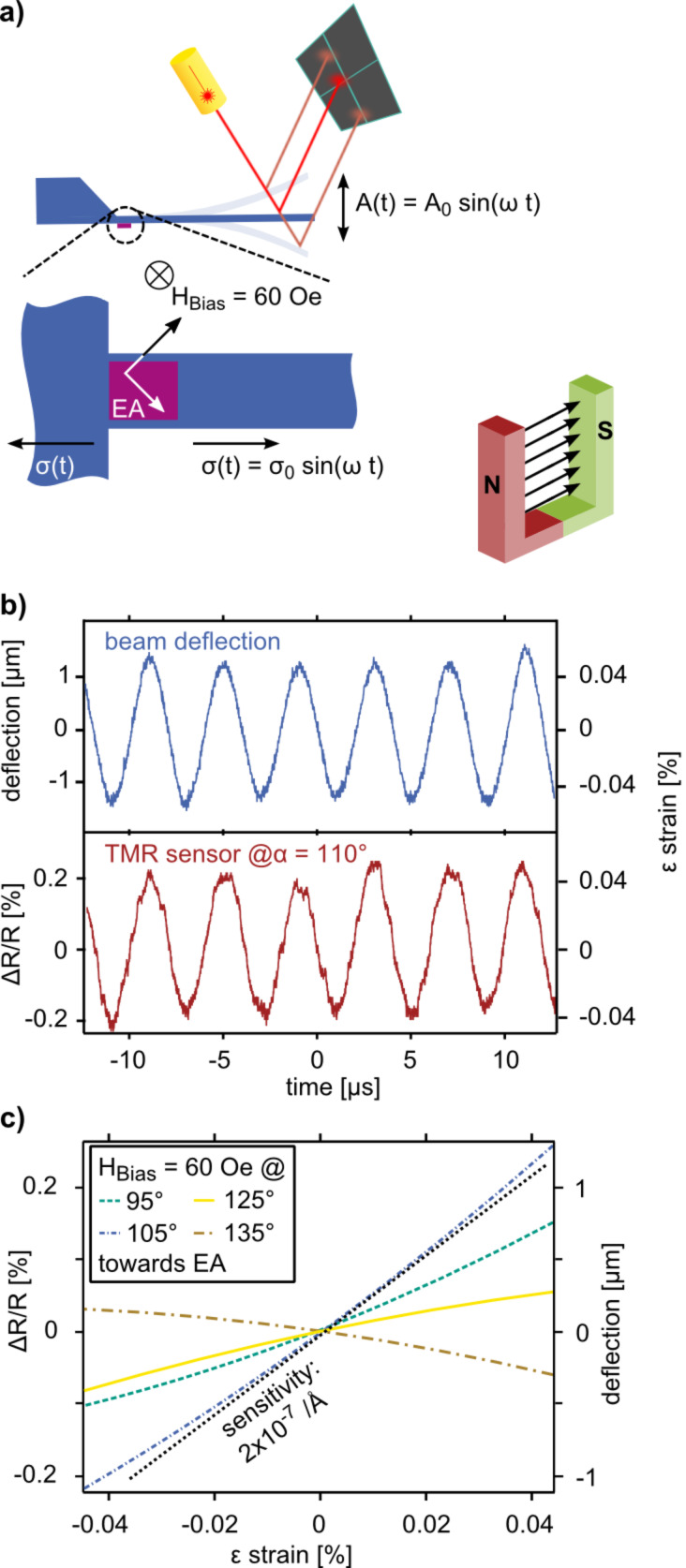
Characterization of AFM cantilevers equipped with strain sensitive TMR sensors. a) The cantilevers deflection can be measured with the beam deflection setup while the strain sensitivity of the TMR sensor can be tuned with a magnetic bias field. b) The oscillation of the cantilever is measured by the beam deflection setup and the strain in the cantilever by the TMR sensor. In this notation, tensile stress corresponds to positive strain. The resistance change of the 27 μm × 27 μm sized TMR sensor can be correlated to the applied strain. c) The resistance change as a function of strain is exemplary plotted for four different angles of the bias field towards the easy axis. The bias field has a strong influence on the strain sensitivity of the TMR sensor.

As shown in Figure 5 the detection principle of a magnetostrictive TMR sensor can be easily applied to measure the bending of an AFM cantilever. In particular, TMR sensors with a CoFeB/MgO/CoFeB magnetic tunnel junction are well known for their very high TMR values [56]. In addition, the use of a Co_40_Fe_40_B_20_ sensing layer leads to high strain sensitivity [57]. Those measurements, however, are done with a 4-point bending apparatus and a magnetic bias field of 60 Oe perpendicular to the magnetization of the pinned reference layer and with tensile stress applied to the junction. On the cantilever level, not only tensile but also compressive stress occurs. The alignment of the initial easy axis of the sensing layer is, therefore, set to 45° against the applied stress. In this way the TMR sensor is sensitive to both compressive and tensile stress what is required for essentially all modes of AFM. Assuming single domain behavior of the two ferromagnetic layers, the conductance of the TMR junction is depending on the angle α between the magnetizations of the two electrodes [58].

To achieve a high resistance change of the TMR junction and a high strain sensitivity, only the sensing layer must be rotated with respect to the reference layer. This can be achieved with the magnetic bias field. The field must be strong enough to rotate the magnetization of the sensing layer but also weak enough to enable strain-induced rotation. We investigated the angular dependence for a magnetic bias field of 60 Oe. The angle α is thereby defined as the angle between the easy axis and the bias field and varies between 0 and 180°. The angle of the bias field was varied in 5° steps while the TMR sensor was saturated along the easy axis between each angle variation. As the setup of our AFM allows for both the measurement of the cantilever deflection by independent means and the response of the TMR sensor as a function of the angle of the magnetic bias field the field can be varied until the optimum is found. The resistance of the 27 μm × 27 μm sized TMR sensor with a resistance area product of 61 kΩ·μm^2^ increases and decreases under the applied tensile and compressive stress, respectively, induced by the oscillation of the cantilever at its resonance frequency (see Figure 5b and Figure 5c). To measure the resistance of the TMR sensor, it is integrated into a Wheatstone bridge configuration with a 20 mV bias voltage. We maintained the voltage drop on the TMR sensor in the unstrained configuration at 10 mV and kept the bridge balanced. The voltage between the midpoints was amplified by 60 dB and low-pass filtered with a cut-off frequency of 400 kHz. This readout was directly fed into a 100 MHz analog–digital converter for recording and comparison with the optical beam deflection readout which is used to measure the deflection of the cantilever. With the deflection, the strain at the base of the cantilever can be approximated by using Hooke’s law and the Young’s modulus of the cantilever beam. In Figure 5c, the sensor response for four chosen field angles is given. The strain sensitivity (slope of the sensor response) varies quite significantly with the incident angle of the magnetic field. The sensor also shows a higher sensitivity for tensile strain (steeper slope for positive values of ε in Figure 5, which can be used in pre-strained junctions or to distinguish between compressive and tensile stress for spectroscopy applications. The TMR junction with a squared geometry used in this work shows the highest strain sensitivity of 2 × 10^−7^ Å, at a bias field angle α of 115° towards the magnetization of the reference layer. For this measurement, we can extract a signal-to-noise ratio of 900 at a bandwidth of 100 kHz which allows one to measure the oscillation of the cantilever on its resonance. For symmetry reasons, the behavior of the TMR sensor can be assumed to have the same sensitivity for negative values of α, however, the signal from the TMR sensor is inverted with respect to the signal for positive values of α.

To investigate the strain sensitivity and the feedback mechanism when using TMR sensors on AFM cantilevers, we fabricated tipless cantilevers and obtained a suitable resolution on gratings [35]. To increase the lateral resolution, however, sharp tips have to be attached to our cantilevers with TMR sensors. By using a combination of focused ion beam and electron beam deposition, tips can be manually been grown on the apex of the cantilever [59]. The use of such tips enables high lateral resolution as tip radii as small as 30 nm can be achieved. The advantage of this approach is that the tip is subsequently grown and without altering the fabrication process of our TMR cantilevers.

**Figure 6:**
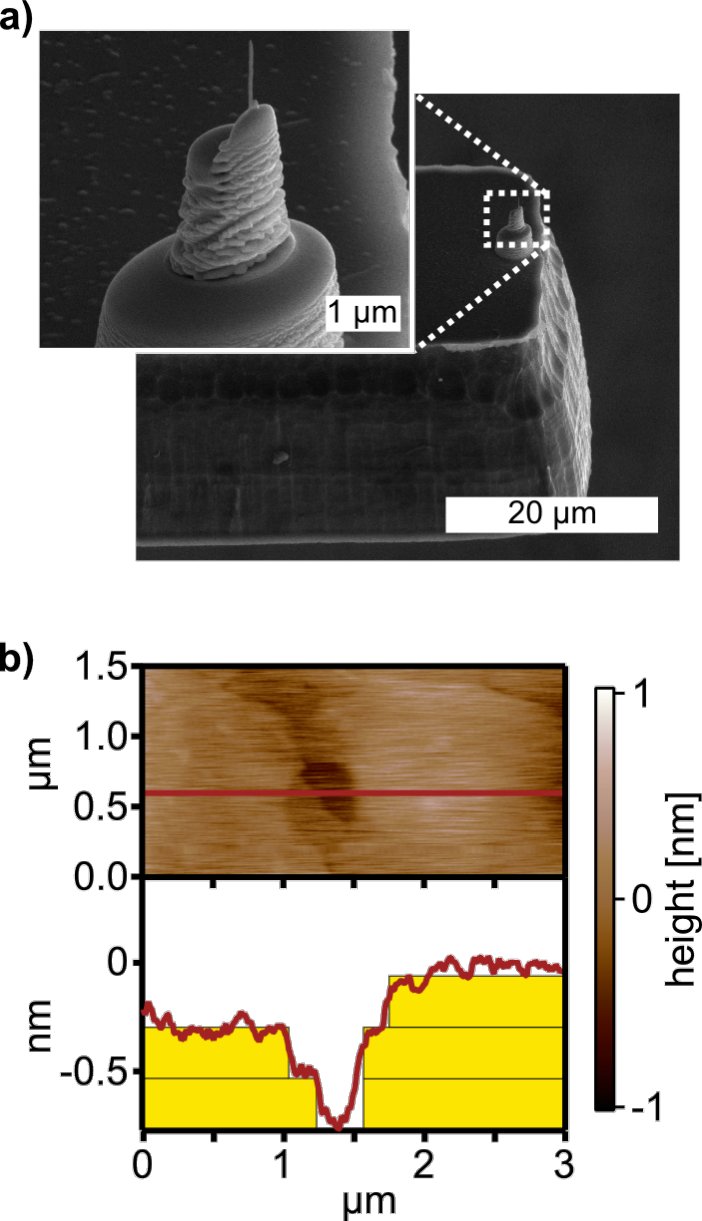
a) To improve lateral resolution, tips with a tip radius of 30 nm were grown by a combination of focused ion beam and electron beam deposition deposition. b) Atomic step-edges on gold(111) terraces can be revealed by amplitude modulation imaging with the feedback on the TMR sensor.

As AFM setups with beam deflection can routinely image smallest features such as atomic step edges, the ability to reveal such features is mandatory to be competitive. Figure 6 demonstrates that atomic-scale resolution can be also obtained with a TMR sensor. The image of atomic step edges on gold(111) was obtained in the amplitude modulation mode in which the cantilever oscillation was detected with the TMR sensor. The applied bias field was chosen for maximum strain sensitivity for the unstrained sensor at 60 Oe and α = 115°. With those parameters, atomic step edges of 2.54 Å height are resolved.

**Figure 7:**
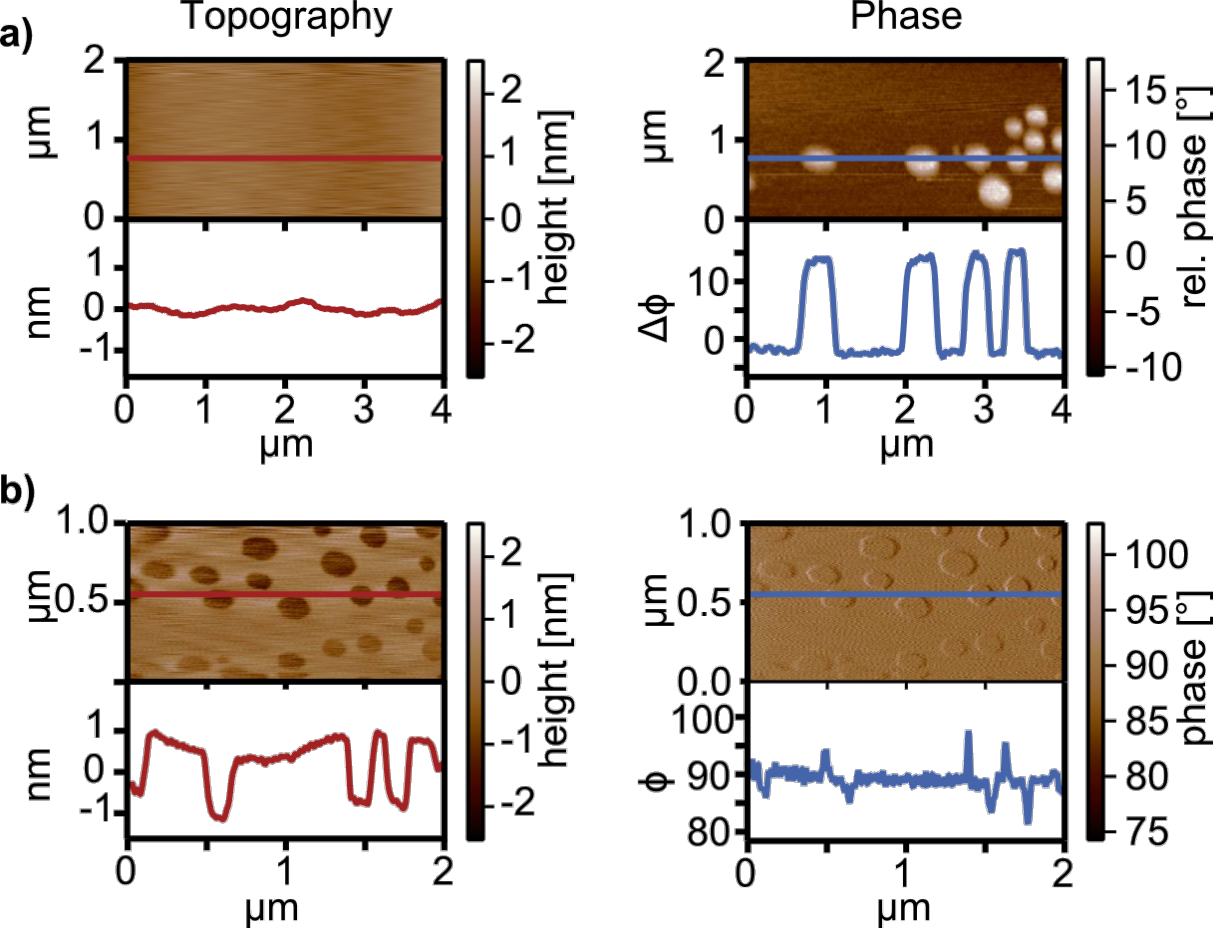
Dynamic mode imaging of FDTS-SAM samples using a TMR sensor with the feedback on amplitude and phase. a) Amplitude modulation mode imaging of FDTS-SAM in SiO*_x_* with a TMR sensor. On this sample system, dissipative tip–sample forces are dominant. Therefore, a high phase-signal contrast can be observed and reveals the different materials of the sample due to different energy dissipation between tip and sample while the amplitude-signal feedback reveals no topographic features. b) On such samples, phase-locked frequency modulation AFM is advantageous and can reveal the topography of the sample. As the cantilevers resonance frequency is fed back to the driving signal by an additional loop, the phase contrast vanishes and is constant at 90°, while the topography with the holes in the SAM is revealed.

For dynamic-mode experiments, the phase-shift signal is of high interest as it provides information about energy dissipation between tip and sample [60–61] and visualizes chemical contrasts [62]. To demonstrate this kind of measurement also with our TMR sensors, we applied polymer blend lithography to pattern structured self-assembled monolayers (SAMs) on hydrophilic SiO*_x_* [63]. In order to obtain a high chemical contrast we used 1.3 nm high monolayers of FDTS (1*H*,1*H*,2*H*,2*H* - perfluorodecyltrichlorosilane), which are well known for their hydrophobicity [64]. If exposed to ambient conditions with a relative humidity of around 40%, the topographic contrast on those two materials disappears in amplitude modulation imaging. The height difference, however, can be observed if the sample is scanned in a liquid [63]. Therefore, we conclude that the vanishing topography contrast in ambient conditions is most likely caused by the thin water films present on hydrophilic SiO*_x_* under ambient conditions [65]. This effect obscures the height difference between the FDTS and SiO*_x_*. However, as shown in Figure 7a, the difference of the energy dissipation between the two materials is observable and the holes in the FDTS-SAM are visible as bright spots in the phase signal. As the phase contrast on this sample system is higher, we altered the feedback and scanned the same sample in a frequency modulation mode [66]. Thereby, the resonance frequency of the cantilever was tracked with a phase-locked-loop (PLL) while its frequency shift was used as a feedback for the topography feedback loop [6]. As the frequency tracking loop feeds back the cantilevers resonance frequency to the driving signal at a 90°phase shift, the contrast in the phase signal disappears as shown Figure 7b. The topography of the sample, however, is revealed clearly (see Figure 7a).

## Conclusion

To conclude, we presented an atomic force microscope with a nested scanner design of two independent piezo scanners for the imaging of surfaces up to 800 × 800 μm^2^. The AFM is capable of switching from the large-area scanner to the small high-resolution scanner. This key feature of the nested scanner design makes the instrument a versatile tool for the analysis of micro- and nanostructures by sequential scanning with both scanners. For the characterization of self-sensing AFM cantilevers based on TMR sensors, the instrument is designed to be operated in externally applied magnetic bias fields to optimize the sensitivity of the TMR sensors. The performance of these sensors has been shown to be sufficient for several operation modes and is capable of imaging smallest feature sizes like atomic step edges.

## Acknowledgments

It is a pleasure to thank Hanaul Noh for fruitful discussions. We acknowledge support by the German Science Foundation DFG (HO 2237/4-2, ME 3616/1-2, RE 1052/31-2). This work was partly carried out with the support of the Karlsruhe Nano Micro Facility (KNMF, http://www.kit.edu/knmf), a Helmholtz Research Infrastructure at Karlsruhe Institute of Technology (KIT, http://www.kit.edu).
